# Patient Stratification for Antibiotic Prescriptions Based on the Bound-Free Phase Detection Immunoassay of C-Reactive Protein in Serum Samples

**DOI:** 10.3390/bios13121009

**Published:** 2023-12-03

**Authors:** Benita Johannsen, Desirée Baumgartner, Michal Karpíšek, David Stejskal, Noémie Boillat-Blanco, José Knüsli, Marcus Panning, Nils Paust, Roland Zengerle, Konstantinos Mitsakakis

**Affiliations:** 1 Hahn-Schickard, Georges-Koehler-Allee 103, 79110 Freiburg, Germany; 2 BioVendor-Laboratorní Medicína a.s., Research & Diagnostic Products Division, Karasek 1767/1, Reckovice, 62100 Brno, Czech Republic; 3 Faculty of Pharmacy, Masaryk University, Palackeho trida 1946/1, 61242 Brno, Czech Republic; 4 Department of Biomedical Sciences, Faculty of Medicine, University of Ostrava, Syllabova 19, 70300 Ostrava, Czech Republic; 5 Institute of Laboratory Diagnostics, University Hospital Ostrava, 17. listopadu 1790/5, 70800 Ostrava, Czech Republic; 6 Service of Infectious Diseases, Lausanne University Hospital, University of Lausanne, Rue du Bugnon 46, 1011 Lausanne, Switzerland; 7 Institute of Virology, Freiburg University Medical Center, Faculty of Medicine, University of Freiburg, Hermann-Herder-Strasse 11, 79104 Freiburg, Germany; 8 Laboratory for MEMS Applications, IMTEK–Department of Microsystems Engineering, University of Freiburg, Georges-Koehler-Allee 103, 79110 Freiburg, Germany

**Keywords:** immunoassay, bound-free phase, C-reactive protein, clinical samples, diagnostics, biomarkers, patient stratification, respiratory tract infections

## Abstract

C-reactive protein is a well-studied host response biomarker, whose diagnostic performance depends on its accurate classification into concentration zones defined by clinical scenario-specific cutoff values. We validated a newly developed, bead-based, bound-free phase detection immunoassay (BFPD-IA) versus a commercial CE-IVD enzyme-linked immunosorbent assay (ELISA) kit and a commercial CE-IVD immunoturbidimetric assay (ITA) kit. The latter was performed on a fully automated DPC Konelab 60i clinical analyzer used in routine diagnosis. We classified 53 samples into concentration zones derived from four different sets of cutoff values that are related to antibiotic prescription scenarios in the case of respiratory tract infections. The agreements between the methods were ELISA/ITA at 87.7%, ELISA/BFPD-IA at 87.3%, and ITA/-BFPD-IA at 93.9%, reaching 98–99% in all cases when considering the calculated relative combined uncertainty of the single measurement of each sample. In a subgroup of 37 samples, which were analyzed for absolute concentration quantification, the scatter plot slopes’ correlations were as follows: ELISA/ITA 1.15, R^2^ = 0.97; BFPD-IA/ELISA 1.12, R^2^ = 0.95; BFPD-IA/ITA 0.95, R^2^ = 0.93. These very good performances and the agreement between BFPD-IA and ITA (routine diagnostic), combined with BFPD-IA’s functional advantages over ITA (and ELISA)—such as quick time to result (~20 min), reduced consumed reagents (only one assay buffer and no washing), few and easy steps, and compatibility with nucleic-acid-amplification instruments—render it a potential approach for a reliable, cost-efficient, evidence-based point-of-care diagnostic test for guiding antibiotic prescriptions.

## 1. Introduction 

Protein biomarkers play a major role in diagnosis, especially in the case of infectious diseases where host response biomarkers may be used in a complementary way with the detection of pathogens and pathogen-specific host immune responses and/or with imaging (ultrasound, radiographic images, and computer tomography), in order to realize a full and reliable clinical assessment [1,2,3,4].

One of the most studied and frequently utilized biomarkers is C-reactive protein (CRP) [5]. In cases of respiratory tract infections (RTIs), for example, CRP is one of the biomarkers whose use has been found to lead to a safe decrease in antibiotic prescriptions [6,7,8,9]. Many clinical trials and guidelines that assess or suggest the use of CRP-guided antibiotics rely on particular CRP concentration threshold levels to provide prescription recommendations [10,11,12,13]. For this reason, well-performing and validated immunoassays for assigning CRP concentrations into specific zones for patient stratification are of key importance. Apart from standard enzyme-linked immunosorbent assays (ELISAs) [14,15], numerous immunoassays have been developed over recent decades. They are at different technology readiness levels, exploit different configurations and detection principles, and exhibit varying advantages regarding speed, sensitivity, multiplexing capacity, and integration compatibility [16,17,18,19,20]. Nevertheless, a generally crucial step in immunoassay development and the roadmap for the transition from laboratories to clinics is the validation of assays using clinical samples and specific clinical scenarios. 

In this context, the authors have developed the bound-free phase detection immunoassay (BFPD-IA), demonstrating its performance with diverse sample matrices and concentration ranges and proving it to hold several competitive advantages that can positively impact its implementation in practice [21]: (i) It has a short incubation duration of 15 min that allows several runs to be conducted during an eight-hour shift. (ii) It has a wash-free nature and capacity for multiplexing in a single reaction well—these enable a reduction in reagent consumption, leading to cost and environmental waste savings. (iii) There is a single incubation step and an easy workflow which render it highly suitable for integration in automated point-of-care (POC) or near-patient systems [22]; additionally, these mean it is potentially attractive for integration into large, laboratory-based systems. (iv) It is compatible with nucleic acid amplification instruments [23]; this offers a unique selling point not only to commercial manufacturers of diagnostic systems but also hospitals, clinics, and general practitioners (GPs). They can save costs when procuring devices, thereby relieving health systems and improving logistics, as they may detect pathogens via nucleic acid detection and quantify protein biomarkers using the BFPD-IA with a single measurement setup.

Due to the promising advantages of the BFPD-IA operating principle, in the current work, we have aimed to assess its potential utility in stratifying patients in specific CRP concentration zones to support decision making regarding antibiotic prescriptions in the case of RTIs. In order to achieve this, we (a) measured the CRP concentration in 53 real clinical samples (serum); (b) classified the measured values into different concentration zones that derive from cutoff values of four representative clinical scenarios for antibiotic prescriptions in the case of RTIs in primary care settings [10,11,12,13,24]; and (c) correlated the acquired results with those of a CE-IVD enzyme-linked immunosorbent assay (ELISA) kit [25] and a CE-IVD immunoturbidimetric assay (ITA) kit [26] on a DPC Konelab 60i clinical analyzer [27] that is routinely used for diagnostic purposes.

## 2. Materials and Methods 

### 2.1. Sample Collection and Ethics Permission

All serum samples were acquired in accordance with the ethics rules laid out in the Declaration of Helsinki and approved by the Ethics Committee of Faculty Hospital Ostrava, Czech Republic. Informed consent was collected for each individual/sample. The medical/collection site was the Institute of Laboratory Diagnostics, University Hospital Ostrava, Czech Republic. The samples were acquired in the framework of Grant TACR/1-24/2019, Program FW-TREND ID: FW01010052: “development of new laboratory tests based on the principle of chemiluminescence analysis on automated platforms for the diagnosis of inflammation, sepsis and cardiovascular diseases”. Therefore, patients experiencing these diverse disease states were the origin of the samples. However, importantly, we did not choose the samples to be tested based on the specific patients’ status; instead, we chose them based on the samples’ CRP concentration values (even if they derive from diverse sources), in order to cover the entire, broad concentration range of primary-care-related RTIs and antibiotic prescriptions (from <20 mg/L to >100 mg/L), for which we intended to test our method. The samples were collected in the hospital and were forwarded to the central laboratory for centrifugation. The samples were stored two hours after collection at −80 °C for further analysis.

### 2.2. Measurements Using the Immunoturbidimetric Assay Kit

CRP values were determined using the immunoturbidimetric assay kit CRP FS (17002) and calibrator TruCal CRP (1 7000 99 10 039) from DiaSys Diagnostic Systems GmbH, Holzheim, Germany, using the instrument DPC Konelab 60i, Thermo Fisher Scientific, Vantaa, Finland. Briefly, the immunoturbidimetric assay kit is a CE-IVD diagnostic reagent kit for quantitative in vitro determination of CRP in serum or plasma on photometric systems. Reagent R1 (250 µL) is pre-incubated together with 15 µL of sample or calibrator for 5 min to reach a temperature of 37 °C. Samples are not pre-diluted and there is no further dilution during the procedure inside the instrument. The reaction starts following the addition of 50 µL of reagent R2. The resulting absorbance is read after an additional 5 min incubation at 37 °C, thereby bringing the total assay time to 10 min. For the calibration of the automated photometric systems, the DiaSys TruCal CRP calibrator set was used in accordance with the kit manufacturer’s protocol [28]. The assigned values of TruCal CRP have been made traceable to the ERM^®^-DA474/IFCC reference material. The measuring range is from 2 to 250 mg/L, and the lower limit of detection is 2 mg/L. 

### 2.3. Measurements Using the CE-IVD ELISA Kit

The sandwich ELISA kit, based on the use of two different mouse monoclonal antibodies for determination of CRP, was obtained from apDia, Turnhout, Belgium (CRP ELISA, 740001, CE-IVD), and the assay was conducted in accordance with the manufacturer’s instructions. The standards used in the kit were calibrated against the NIBSC International Standard 85/506. The kit has been validated using a commercial assay by the manufacturer (CRP Vario, Sentinel Diagnostics, Milano, Italy). Briefly, serum samples and calibrators were diluted 1:1000 in two steps and then incubated for 30 min in the plate coated with specific antibody. After a washing step (5 min), an anti-CRP antibody labelled with horseradish peroxidase (HRP) was added and the mixture was incubated for a further 30 min. After removing the unbound conjugate during a 5 min washing step, the microtiter plate was incubated for 10 min with a chromogen solution containing tetramethylbenzidine and hydrogen peroxide to develop a blue color. Thus, the total time was 80 min. The enzymatic reaction was stopped through the addition of 0.5 M H_2_SO_4_ and the absorbance values were determined at 450 nm (ELx808, BioTek, Winooski, VT, USA). The measuring range is 5–100 mg/L, and lower limit of detection is 0.59 mg/L. Samples exceeding the measurement range were measured after a 1:2000 dilution, instead of the 1:1000 dilution that the other samples had.

### 2.4. Measurements Using the BFPD-IA

**Figure 1:**
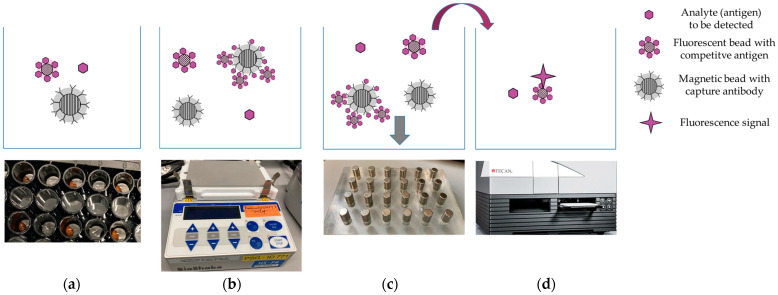
Schematic workflow of BFPD-IA: (**a**) mixing of all reagents, namely the serum sample, the assay buffer, the antibody-coated magnetic beads, and the competitive-antigen-coated fluorescent beads in a reaction well of a microtiter plate (MTP); (**b**) incubation of the reagents in a single step, on a BioShake iQ device (QInstruments, Jena, Germany) (37 °C, 650 rpm), which leads to the capturing of the sample antigens and the fluorescent-bead-coupled antigens on the antibody-coated magnetic beads; (**c**) use of a custom-made magnet rack, positioned under the MTP for magnet-induced separation and collection of the magnetic beads at the bottom of each MTP well (the gray arrow indicates the collection of magnetic beads); (**d**) transfer of 50 µL of supernatant (bound-free phase) to another well for fluorescence detection on Tecan Spark M10 Tecan (Männedorf, Switzerland)—the transfer is indicated with the magenta-colored arrow.

The operating principle of the BFPD-IA is described in detail in a paper by Johannsen et al. [21]. The competitive format of the CRP BFPD-IA includes the sample containing the analyte, an assay buffer, capture-antibody-coated magnetic beads (MBs), and competitive-antigen-coated fluorescent beads (FBs). These are mixed in a single reaction/incubation step followed by the separation of the magnetic beads and the measurement of the unbound fluorescent beads (supernatant) (Figure 1).

The 2.8 μm diameter, tosyl-activated magnetic beads (Dynabeads, M-280) and the 200 nm diameter carboxyl-activated fluorescent beads (F8810, red (excitation 580 nm/emission 605 nm)) were both purchased from Thermo Fisher Scientific, Waltham, MA, USA. The MBs were coated with anti-human CRP antibodies (A80-125A, Fortis Life Sciences (Bethyl), Waltham, MA, USA) and the FBs were coated with native CRP protein (CAS 9007-41-4, Sigma-Aldrich, Darmstadt, Germany) in accordance with protocols described in previous work [21,22]. The assay buffer (dilution buffer, MDB) was provided by BioVendor, Brno, Czech Republic. 

A total volume of 75 µL consisting of the sample or standard (2.5 µL), the assay buffer (63 µL), the MBs (5 µL of 20 mg/mL stock), and the FBs (4.5 µL of initial 20 mg/mL stock diluted 1:10 prior to the addition to the reaction) was pipetted into a microtiter well plate (96 wells, polystyrene, non-binding, Greiner Bio-One, Frickenhausen, Austria) and incubated on a BioShake iQ device (QInstruments, Jena, Germany) at 650 rpm for 15 min at 37 °C. Afterwards, the plate was positioned over an in-house-fabricated magnet rack for rapid (maximum 1 min) MBs collection and 50 μL of the supernatant was transferred to another well. The readout was then performed on a Spark M10, Tecan (Männedorf, Switzerland), using monochromatic filters at 569 nm for excitation and 614 nm for emission. Notably, the same batch of antibody-coupled MBs and the same batch of competitive antigen-coupled FBs were used throughout the study, both for the standard curves and for the serum samples measurements. The coupled beads were stored at +4 °C and all experiments lasted a few days—a timeframe that the authors have detailed in an earlier work [21]; this ensured that the beads and the coupled antibodies/antigens do not lose activity when they are stored in liquid form (as used now) or when they are stored in air-dry form in a microfluidic cartridge [22], when measuring a CRP Certified Reference Material (CRM).

For the standard curve, the standards were created by spiking the following known concentrations of human CRP native antigen (CAS 9007-41-4, Sigma-Aldrich, Darmstadt, Germany) in CRP-free human serum (HyTest, Turku, Finland): 0 (negative control), 15, 30, 60, 90, 115 mg/L. Each measurement plate included one or two full sets of standards, which is a common procedure that is also performed in central labs. Four plates and six individual standard curves were run in total. Out of these, the average standard curve was calculated, so that each standard concentration is an average of N = 6 measurements (raw values in Supplementary File S1). The non-linear curve fit of the average calibration curve was acquired using a four-parameter sigmoidal equation (Supplementary File S1) and OriginPro 2019 (64-bit, v. 9.6.0.172) software. Based on the fitting, the limit of blank (LOB) and the limit of detection (LOD) were calculated at 6.3 and 11.0 mg/L, respectively; these are well below the lowest cutoff value of 20 mg/L that applies to all four examined scenarios.

Each of the 53 samples was measured once, which is also in line with the handling protocols of central laboratories, diagnostic centers, and hospitals (e.g., the standard workflow in the partner Lausanne University Hospital). In a secondary analysis, we calculated the relative combined uncertainty of a single measurement of an unknown sample. This was calculated for BFPD-IA based on the inter-day variation in the standards, as follows (we expect this standards-based calculation of uncertainty to be quite representative also for the unknown serum samples because the matrix used for the standard was CRP-free serum, spiked with the standards concentrations): First, each of the six relative fluorescence unit (RFU) values for each standard concentration was inserted in the four-parameter standard curve fit (Supplementary File S1), thereby deriving the table in Supplementary File S2. Then, the average, standard deviation, and coefficient of variation CV (%) of these six concentration values were calculated. Eventually, the average of these CVs was taken, providing the range that the measured value of each unknown sample is likely to fall into, with a probability of 65% (±1 SD). For the BFPD-IA, this was calculated to be 6.4%. For the ELISA and ITA, the values are provided by the manufacturer to be 10.1% and 3.3%, respectively. When a sample was measured below the LOD or above the highest standard concentration (115 mg/L), this sample was taken into account only for the qualitative analysis (Section 3.2). For this quantitative analysis (Section 3.1), a total of N = 37 samples were included.

Finally, we visually inspected each sample’s color before measuring to ensure that we were including non-hemolyzed samples, in line with recommendations (see screenshot of the blood vials in Supplementary File S3).

## 3. Results and Discussion

### 3.1. Methods Comparison

**Figure 2:**
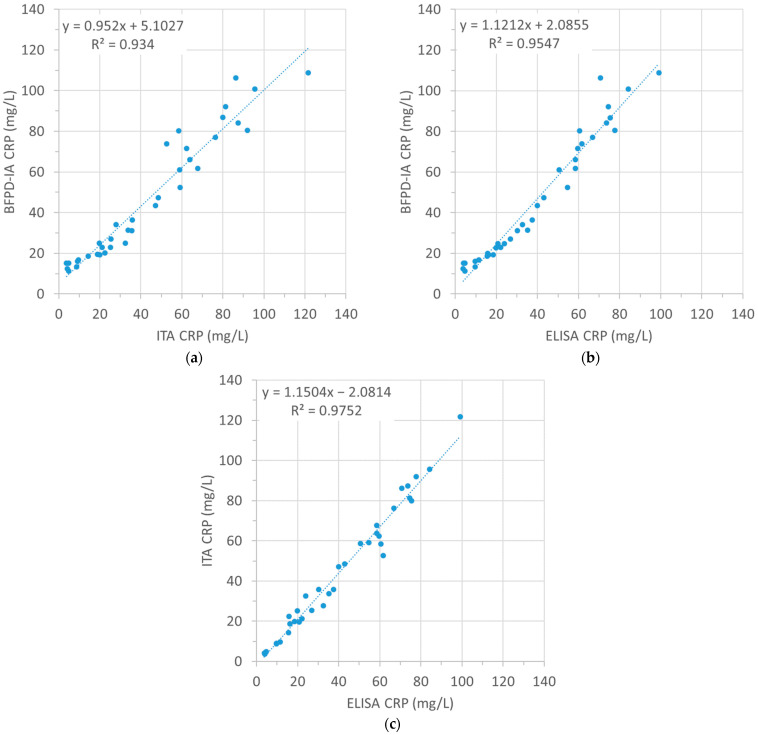
Scatter plots with the linear fit indicating the correlation of CRP concentration measurements between (**a**) BFPD-IA and ITA, (**b**) BFPD-IA and ELISA, and (**c**) ELISA and ITA. N = 37 (out of the total 53) samples are shown in all graphs, as this was the number of samples that was quantifiable within the detection limits of BFPD-IA.

For the BFPD-IA calculation of the CRP concentration, we used the average standard curve (Supplementary File S1), which we acquired as described in Section 2.4. As it was the average of six individual curves over four measurement days, it is representative of the inter-day and inter-plate variability. The average signal CV over all the standards was 6.6% (7.3% when including the negative control). Furthermore, our quantification range (11–115 mg/L) is comparable with those used by some commercial systems [29,30]. The CRP concentrations of the clinical samples are given in Supplementary File S4. We correlated the absolute values acquired using the three measurement methods by means of scatter plots (Figure 2). 

Based on the deviation of the slopes from the absolute y = x, we observe that there is closeness in all three cases. The correlation between BFPD-IA and ITA (slope 0.95, thereby deviating by only 5% from y = x) is somewhat higher than between BFPD-IA and ELISA (slope 1.12, thereby deviating by 12% from y = x), as well as higher than that between the ITA and ELISA (slope 1.15, thereby deviating by 15% from y = x). This raises interesting discussion points about the possible reasons for the discrepancies between immunoassay measurement methods.

Discrepancies are generally expected even between commercial systems when measuring the same samples [31]. Previous studies by Minnaard et al. confirm that there can be considerable variation between different CRP POC tests and a laboratory reference standard [29]. This can be attributed to diverse factors like different detection principles, assay formats (homogeneous, heterogeneous, sandwich, and competitive), and the inherent characteristics of the assay components themselves, such as the association and dissociation kinetics, if the assay is conducted using different antibodies (e.g., monoclonal or polyclonal), or if the antibodies recognize different epitopes; this has been reported to be at least partly responsible for different results [29,32,33,34,35,36]. 

Specifically in our case, the ELISA kit used monoclonal antibodies while the ITA kit and BFPD-IA used polyclonal (all from different suppliers). Even when the same platform is used with different dilution buffers, or when the same antibody–antigen pairs are used on different platforms, the outcomes may differ. Another source of deviation may be that ELISA requires two pre-dilution steps (in total 1000×) of the sample before the assay, while ITA and BFPD-IA do not (Section 2). In terms of assay format, the used ELISA kit was designed as a sandwich assay to be performed on a standard microtiter plate (MTP), followed by a fluorescence readout. This means that it includes some manual steps, but also depends on the fluorescence readout equipment used after assay incubation, whereas the ITA was performed in a closed, fully automated clinical analyzer system used in routine diagnosis. Furthermore, the surface of an MTP well has a limited capacity for binding antibodies (Maxisop: 0.6 µg/well in a 1.0 cm^2^ well surface used in this ELISA [37], or less, e.g., for example in Polysorp or Costar High Binding). In contrast, the ITA is a homogeneous immunoassay format and therefore has no such limitations. These elements explain the deviation of 15% from the y = x in the ELISA vs. ITA scatter plot in Figure 2c. The latter argument can also justify the closeness between the BFPD-IA and ITA, since the BFPD-IA, like the ITA, is performed in liquid. In addition, the incubation time in BFPD-IA (~15 min for CRP) is much closer to that of ITA (~10 min) than to that of ELISA (~80 min, plus more than 60 min to pre-dilute the samples, 1000× in two steps). That is why the slope in the BFPD-IA vs. ITA scatter plot in Figure 2b deviates from the y = x by only 5%. Lastly, the fact that the collection of samples that we tested evenly covered the entire desired range from <20 mg/L to >100 mg/L, and without bias or data accumulation around high or low values, offers an increased validity to the aforementioned correlation between BFPD-IA, ITA, and ELISA.

Last but not least, the quality of the clinical samples that are used for performance characterization of a new method, and immunoassays in general, is very crucial. In particular, it has been reported that hemolyzed samples can interfere with measurements due to the destruction of red blood cells, which leads to the release of substances like hemoglobin that are strongly related to the sample’s optical properties [38,39]. In vitro hemolysis itself may derive from sub-optimal pre-analytical sample management, including blood drawing, handling, transportation, storage, and preparation for testing [40]. Therefore, for many determination methods that are based on the optical properties of the serum or plasma sample (e.g., ELISA and ELISA-like immunoassays such as the BFPD-IA), hemolyzed samples should not be measured, as they can lead to false results [41,42,43]. Supplementary File S3a shows some examples of serum colors, based on which one can judge whether the sample is suitable for use or not [44]. The figures in Supplementary File S3b show some of the samples used in our study. We encountered no hemolyzed samples in our collection. Possible icteric samples (based on color assessment, Supplementary File S3c) could nevertheless be measured using all assays.

### 3.2. The Clinical Relevance

Taking for granted that deviations in the absolute quantification between measurement methods will exist, an important question follows: how crucial is the absolute CRP quantification itself, in terms of its clinical utility as a biomarker for antibiotic prescription decision support? From a clinical perspective, and for biomarkers like CRP—but also others like procalcitonin—randomized controlled studies have shown that it is efficient to stratify patients in ranges (zones) defined above or below concentration cutoff values to guide antibiotic prescriptions [10,11,12,13,45,46]. The cutoffs and ranges may differ depending on the clinical condition (e.g., RTIs, tropical infections, and sepsis), age group (elderly versus children or infants), healthcare settings (primary care, emergency unit, and intensive care unit), etc. [47]. However, in general, the concept that semi-quantification is sufficient over absolute quantification remains valid. Furthermore, the utilization of concentration cutoff values and classification zones between them can also help simplify the immunoassay development (avoid over-engineering), as developers can skip developing CRP immunoassays covering more orders of magnitude than required [29].

This zone-based clinical assessment drove us to perform such a semi-quantitative analysis, in addition to the previous quantitative methods comparison, in order to address the clinical perspective. We considered four clinical scenarios as described below, whose cutoff values and corresponding concentration zones are summarized in Table 1:

- Scenario 1: Lower and upper respiratory infections at the GPs [10].
- Scenario 2: Exacerbations of chronic obstructive pulmonary disease (COPD) at the GPs [11].
- Scenario 3: Lower respiratory infections in an elderly care home [12].
- Scenario 4: Cutoff values in national guidelines for antibiotic prescriptions in GP practices and emergency units in the UK and the Netherlands [13,24].

We chose these scenarios for the following reasons: (i) because they proved to be safe and efficient in terms of antibiotics reduction in clinical trials or they are recommended in guidelines; (ii) because they represent the primary care settings where we first intend to implement our method, since CRP testing in primary care is widely used and recommended. These scenarios are frequently encountered in GP practices and nursing homes, where diagnostic tests with clear guidance are urgently needed to decrease inappropriate antibiotic prescriptions. Usually, the recommendation is to not prescribe antibiotics to patients with biomarker concentrations within the lower zone, and to prescribe to those with concentrations within the higher zone. There is often an intermediate zone in which it is recommended to integrate the biomarker concentration with the clinical evaluation of the patient.

Based on these scenarios, we classified each sample in one concentration zone. This classification is shown in Supplementary File S4. For the qualitative analysis of this clinical perspective section, we took into consideration all 53 samples, since even those that were not absolutely quantified in Section 3.1 could still be assigned to concentration zones. Based on this analysis, we could determine how many of the 53 samples were classified using BFPD-IA in the same concentration zone as those classified using ITA and ELISA, but also through ELISA versus ITA, for each of the four scenarios (Table 2). The results indicate the very close potential agreement between BFPD-IA and ITA in all examined scenarios. This is also in accordance with the quantitative results of Figure 2.

**Figure 3:**
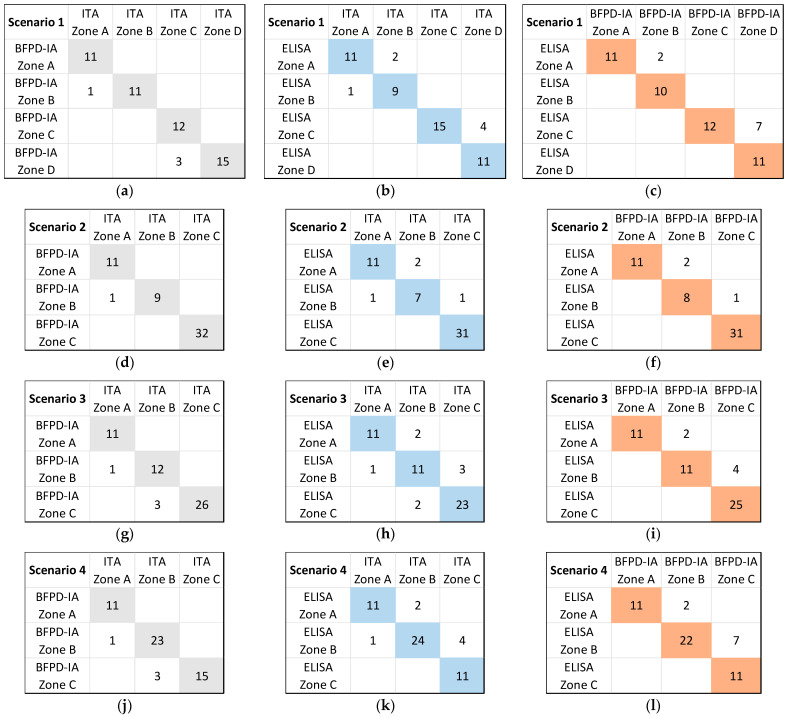
Number of samples classified using ITA, ELISA, and BFPD-IA in defined concentration zones per scenario: (**a**) scenario 1, comparison of ITA vs. BFPD-IA; (**b**) scenario 1, comparison of ITA vs. ELISA; (**c**) scenario 1, comparison of BFPD-IA vs. ELISA; (**d**) scenario 2, comparison of ITA vs. BFPD-IA; (**e**) scenario 2, comparison of ITA vs. ELISA; (**f**) scenario 2, comparison of BFPD-IA vs. ELISA; (**g**) scenario 3, comparison of ITA vs. BFPD-IA; (**h**) scenario 3, comparison of ITA vs. ELISA; (**i**) scenario 3, comparison of BFPD-IA vs. ELISA; (**j**) scenario 4, comparison of ITA vs. BFPD-IA; (**k**) scenario 4, comparison of ITA vs. ELISA; (**l**) scenario 4, comparison of BFPD-IA vs. ELISA. Such representation allows us to note the relative discrepancy between two methods and towards which zone.

Notably, the results of Table 2 are not zone-specific and thus do not reveal which zone(s) the discrepancies appear in. Therefore, we examined whether there was any particular zone within each scenario that was more prone to discrepancies. For this purpose, we considered the ITA-generated zone classification as the “true” one, since this is performed on a fully automated commercial system used in routine diagnostic practice. We then created Figure 3 by using a comparison style similar to that used elsewhere in the literature [35]. For all four scenarios, we show the agreement or deviation between specific zones. Generally, the discrepancies between methods do not seem to be zone-specific. This “zone-independent” performance of BFPD-IA enhances its potential to be used reliably in several different scenarios and concentration zones. It is also important to note that any existing discrepancies appeared between “neighboring” zones, and not, e.g., between Zone C (typically for prescribing antibiotics) and Zone A (typically for not prescribing), or vice versa.

As mentioned in Section 2.4, in a secondary analysis, we considered that the single measurement of an unknown sample can be accompanied by a relative combined uncertainty, being 10.1% for ELISA, 3.3% for ITA, and 6.4% for BFPD-IA (the former two provided by the supplier, the latter calculated using the method described in Section 2.4 and Supplementary File S2). The slightly higher value for ELISA, as compared to BFPD-IA and ITA, lies in the fact that it requires two pre-dilution steps of the sample prior to the actual assay (Section 2.3), which adds to the relevant uncertainty. This is one advantage of BFPD-IA—it requires no sample pre-dilution prior to the actual assay and the said sample can be used as such, undiluted. In all the three methods that we used, the uncertainties were lower than the 15% that was reported by Minnaard et al. through an analytical performance comparison between five POC CRP tests [29].

Based on the relative combined uncertainty of the secondary analysis, we carried out correlations similar to the ones shown in Figure 3 and summarized them in Supplementary File S5, including additional options for samples that may potentially fall in either one of two neighboring zones. Such cases are marked as zones in brackets in Supplementary File S4 and we consider that a sample classified in Zone X and potentially in Zone Y (marked as ‘X(Y)’) is in agreement with a sample classified in Zone Y and potentially in Zone X (marked as ‘Y(X)’). Thus, we consider samples classified as X, X(Y), or Y(X) as potentially being in agreement. With such broader, supplementary analysis, the agreement between the three methods reaches the 98–99% (the only few outliers are shown outside the colored diagonal boxes in Supplementary File S5). This secondary analysis also leads to the generation of error bars in each measured data point in the scatter plots, which represent this relative combined uncertainty (Supplementary File S6).

Lastly, some limitations of our study were as follows: (i) the number of samples, which would not be sufficient to conduct a strictly defined clinical study in each of the aforementioned four scenarios; (ii) the fact that these samples did not derive from the specific population of the four scenarios but from diverse sources (see also Section 2.1) and were used to “simulate/extrapolate” the situation of these scenarios and to cover the entire range of concentrations that we needed to test, irrespective of the origin of the samples; and (iii) the fact that each serum sample was measured once, instead of in triplicate, which led to the calculation of the relative combined uncertainty (Section 2.4 and Supplementary File S2) based on the standards only and without having the contribution of the serum samples in that uncertainty.

### 3.3. The Implementation Perspective and Integration in Clinical Practice

In the broad perspective of infection diagnosis and antibiotic prescriptions in real clinical practice, one needs to always keep in mind that the CRP values (and any biomarker values generally) act only as a support to the final decision-making clinicians, who eventually need to co-assess the CRP value together with their clinical evaluation of the patient and other tests, such as imaging and microbiological diagnostics [46,48,49,50,51]. The usefulness of such complementary diagnostics is evident through recent trials with integrated biomarker values into clinical decision-support tools to provide support/guidance in decision making for prescribing antibiotics [10,46,52,53,54,55,56]. In such cases, the entry value of CRP concentration into the algorithm is a single number without including any range of relative combined uncertainty. This is why the core of our analysis was accordingly conducted with single measurement values, i.e., in line with the diagnostic practice. In addition, this value will not be “hidden” in the algorithm’s “black box”; instead, it will be made available to the clinician. Therefore, concentrations measured just below or just above the cutoff values (e.g., 99 versus 101 mg/L) may likely be handled in a similar manner by the clinician.

Ultimately, whether and how a clinician will adopt and make use of available diagnostic tests and algorithms depends on their integrability into the clinical process and on several other factors, including clinical presentation, co-morbidities, the clinician’s own experience, the concordance of the biomarker results with other diagnostic tests, trust in new technologies, and—of course—the reliability of the technology itself [57,58,59]. Regarding this last aspect, and from the performance perspective, the BFPD-IA proved itself to be a valuable, trustworthy immunoassay technology, performing comparably to ITA on the fully automated DPC Konelab 60i clinical analyzer used in routine diagnosis. In addition, the BFPD-IA workflow has some competitive advantages, such as (i) the quick results, allowing several runs to be performed in an eight-hour shift; (ii) the simplicity of workflow, being wash-free with only a few simple steps, cutting both financial and environmental waste down significantly, in contrast to the typically several washing steps of ELISA; (iii) the fact that the sample can be used without pre-dilution, thereby preventing additional dilution-induced errors. These features can render BFPD-IA attractive for integration as a kit in high-throughput systems for central laboratories or in POC or near-patient systems. In fact, the latter option has already been explored successfully, and proven to be compatible even with PCR-performing instruments (i.e., a CRP-ImmunoDisk [22] and a PCR-RespiDisk [60] cartridge on the same processing device), thereby broadening the fields of applicability and implementation. Naturally, before positioning our assay as a potential diagnostic candidate in a POC tool in primary care, the method needs to be tested according to IVDR.

## 4. Conclusions

In this work, we evaluated the capability of a bead-based, bound-free phase detection immunoassay to quantify CRP concentration in serum clinical samples, which we stratified in specific concentration zones of RTI-relevant clinical scenarios. We also conducted this assessment with one CE-IVD ELISA kit and one CE-IVD ITA kit, the latter performed on the fully automated DPC Konelab 60i clinical analyzer, used in routine diagnosis.

The comparison of the methods through absolute CRP quantification in 37 samples and scatter plot slopes revealed the following agreements: 95% between BFPD-IA and ITA (slope 0.95), 88% between BFPD-IA and ELISA (slope 1.12), and 85% between ITA and ELISA (slope 1.15). The measurement range of the BFPD-IA (11–115 mg/L) was sufficient to classify the samples in the clinically relevant concentration zones. The clinical comparison performed through a semi-quantitative analysis of 53 samples and classification in four representative clinical scenarios revealed the following average agreements: 93.9% between BFPD-IA and ITA, 87.3% between BFPD-IA and ELISA, and 87.7% between ITA and ELISA. The relative combined uncertainty when measuring the unknown samples was calculated at 10.1% for ELISA, 6.4% for BFPD-IA, and 3.3% for ITA. 

We attributed the overall closeness between ITA and BFPD-IA and their differences from ELISA to their operating principles, namely the fact that the former two are assay formats performed in liquid, without pre-analytical dilution of the sample and in similar assay timeframes, while ELISA is performed on a microtiter plate through surface-immobilized antibodies. The only small deviation between BFPD-IA and ITA was attributed to the manual operation of the former versus the fully automated operation of the latter. The time-to-result was 20 min for BFPD-IA; this is of the same order of magnitude as the fully automated ITA on the DPC Konelab 60i clinical analyzer (10 min) and is 4× faster than the ELISA (80 min). Furthermore, BFPD-IA and ITA did not require pre-analytic dilution of the sample, while the ELISA kit required a 1:1000 (or, sometimes, 1:2000) pre-dilution.

Such analytical agreement between the BFPD-IA and the routine diagnostic ITA on the DPC Konelab 60i clinical analyzer, together with some inherent structural and functional advantages of the BFPD-IA—such as the few wash-free and dilution-free assay steps, the ease of performance, the reduced reagent consumption, the quick time to result, and the versatile integration and automation potential—can render the BFPD-IA a potential approach for the detection of CRP in clinical scenarios that are relevant to decision making regarding antibiotic prescriptions in RTIs. Thus, our results justify and encourage follow-up work on clinical validation on a larger scale, with diverse patient groups and further clinical scenarios, in combination with other protein biomarker and/or molecular diagnostics and clinical algorithms.

## Figures and Tables

**Table 1:** Zones between cutoff concentrations (mg/L) for the four examined scenarios. In zones marked green, the recommendation is to not prescribe antibiotics. In zones marked yellow, the recommendation is to co-assess with further clinical signs, or even molecular diagnostics, as these are considered “clinically grey zones”. In zones marked red, the recommendation is to prescribe antibiotics.

Zones between Cutoff Concentrations	Scenario 1	Scenario 2	Scenario 3	Scenario 4
Zone (A)	≤20.0	≤20.0	≤20.0	≤20.0
Zone (B)	20.1–50.0	20.1–40.0	20.1–60.0	20.1–99.9
Zone (C)	50.1–99.9	>40.0	>60.0	≥100.0
Zone (D)	≥100.0			

**Table 2:** Agreement in zone classification between combinations of the used measurement methods, expressed as “(expected agreement-disagreement)/(expected agreement)”. Details are provided in Supplementary File S4.

	Scenario 1	Scenario 2	Scenario 3	Scenario 4
Agreement between ITA and ELISA, considering ITA as the reference of ELISA (average 87.7%)	46/53 (86.8%)	49/53 (92.5%)	45/53 (84.9%)	46/53 (86.8%)
Agreement between BFPD-IA and ELISA, considering ELISA as the reference of BFPD-IA (average 87.3%)	44/53 (83.0%)	50/53 (94.3%)	47/53 (88.7%)	44/53 (83.0%)
Agreement between BFPD-IA and ITA, considering ITA as the reference of BFPD-IA (average 93.9%)	49/53 (92.5%)	52/53 (98.1%)	49/53 (92.5%)	49/53 (92.5%)

## Data Availability

Data are contained within the article or in the Supplementary Materials.

## Supplementary Materials

The following supporting information can be downloaded at: https://www.mdpi.com/article/10.3390/bios13121009/s1, File S1: Standard curves for the BFPD-IA; File S2: Calculation of relative combined uncertainty for BFPD-IA; File S3: Quality of samples; File S4: CRP concentrations measured using BFPD-IA, ELISA, and ITA, and classification into zones; File S5: Extended tables of correlation between methods and for each concentration zone; File S6: Secondary analysis of the scatter plots including the relative combined uncertainty.
