# Supplementary material for: Patient Stratification for Antibiotic Prescriptions Based on the Bound-Free Phase Detection Immunoassay of C-Reactive Protein in Serum Samples
Source: Biosensors (Basel). 2023 Dec 3;13(12):1009. doi: 10.3390/bios13121009 (PMC10741775; doi:10.3390/bios13121009)
Supplement: Supplementary file 1 [file biosensors-13-01009-s001.zip › biosensors-2705916-supplementary.pdf]

biosensors

MDPI

Supplementary Materials

Patient Stratification for Antibiotic Prescriptions Based on the Bound-Free Phase Detection Immunoassay of C-Reactive Protein in Serum Samples

File S1. Standard curves for the BFPD-IA.

(**a**) CRP standards concentration values (in mg/L) in each of the six standard curves performed in four different microtiter plates (correspondingly in four days), along with the average, standard deviation, and coefficient of variation (CV, representing the inter-assay variation) of the measured signal (RFU: "Relative Fluorescence Units").

CRP standards concentration (mg/L)	Day 1 Plate 1 curve 1 (RFU)	Day 1 Plate 1 curve 2 (RFU)	Day 2 Plate 2 curve 3 (RFU)	Day 2 Plate 2 curve 4 (RFU)	Day 3 Plate 3 curve 5 (RFU)	Day 4 Plate 4 curve 6 (RFU)	AVERAGE (RFU)	STDEV (RFU)	CV (%)
0	660	819	824	922	778	810	802	85	10.6%
15	1899	2066	1821	1918	1762	1659	1854	140	7.6%
30	4363	4750	4248	4102	4299	3741	4250	330	7.8%
60	18097	18441	18686	18005	19814	19186	18705	692	3.7%
90	26958	30247	28149	31520	30869	27688	29239	1880	6.4%
115	34132	31199	35939	35226	30935	30013	32907	2500	7.6%

(**b**) Average standard curve acquired after averaging the six individual standard curves.

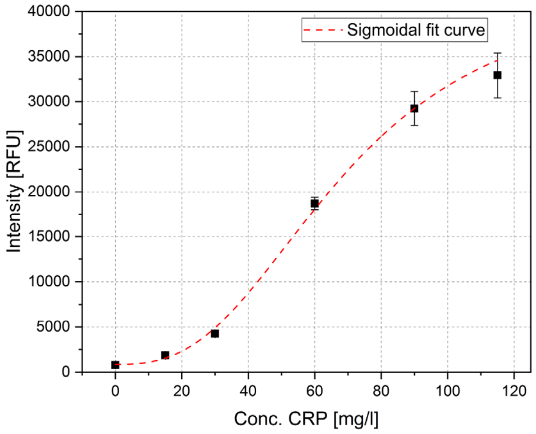

(**c**) Four-parameter sigmoidal curve fit performed on the aforementioned average standard curve, for calculating the CRP concentrations of the clinical samples. The values of the fitting are given in the table below.

$$x = x_0 \sqrt[p]{\frac{y - A_1}{A_2 - y}}$$

Parameter	Value of calibration curve
A_1	876.30226 ± 188.9493
A_2	43050.26242 ± 11673.1537
x_0	68.92887 ± 15.0621
p	2.70009 ± 0.42703

(**d**) Equations for the calculation of limit of blank (LOB) and limit of detection (LOD):

$$LOB = Intensity_{blank} + (1.645 * standard\ deviation_{blank})$$

$$LOD = LOB + (1.645 * standard\ deviation_{lowest conc.})$$

File S2. Calculation of relative combined uncertainty for BFPD-IA.

The rationale for calculation of the relative combined uncertainty for BFPD-IA single measurements of serum samples is as follows:

The relative combined uncertainty, $u_{c,rel}$, is intended to include diverse sources of uncertainties and is given by the equation:

$$u_{c,rel} = \sqrt{u_{1,rel}^2 + u_{2,rel}^2 + u_{3,rel}^2 + u_{4,rel}^2}$$

The four types of uncertainty that are considered the most important for our analysis are:

- uncertainty from standards, $u_{1,rel}$
- uncertainty from averaging real (serum) samples measured in triplicates, $u_{2,rel}$
- uncertainty of the measurement device, $u_{3,rel}$
- uncertainty of pipetting, $u_{4,rel}$

In our work, because we measured the standards in different microtiter plates and on different days, such inter-day uncertainty contains inherently the variations of the measurement device and the pipette, so the $u_{3,rel}$ and $u_{4,rel}$ are already considered and included in the $u_{1,rel}$; that is why they are not included in the square root function, otherwise we would have applied them twice. Thus:

$$u_{c,rel} = \sqrt{u_{1,rel}^2 + u_{2,rel}^2}$$

As we measured each sample once and not in triplicate (see in manuscript Section 3.2 about limitations of the study), we cannot calculate the quantity $u_{2,rel}$. Therefore, it remains that:

$$u_{c,rel} = \sqrt{u_{1,rel}^2} \rightarrow u_{c,rel} = u_{1,rel}$$

In order to calculate the $u_{1,rel}$, we apply a common methodology, where we refer to CVs based on concentrations and not on signal (RFUs) that is described in Section 2.4 and the values are given in the below table. Eventually the relative combined uncertainty for BFPD-IA (which, in our case, refers to the relative uncertainty for standards) is 6.4% for the BFPD-IA.

**Day 1 Plate 1 curve 1 (mg/L)**	**Day 1 Plate 1 curve 2 (mg/L)**	**Day 2 Plate 2 curve 3 (mg/L)**	**Day 2 Plate 2 curve 4 (mg/L)**	**Day 3 Plate 3 curve 5 (mg/L)**	**Day 4 Plate 4 curve 6 (mg/L)**	**AVERAGE (mg/L)**	**STDEV (mg/L)**	**CV (%)**
n/a	n/a	n/a	n/a	n/a	n/a	n/a	n/a	n/a
17.5	18.6	17.0	17.7	16.6	15.9	17.2	0.9	5.5%
28.3	29.5	27.9	27.4	28.1	26.1	27.9	1.1	4.0%
60.1	60.8	61.4	59.9	63.9	62.5	61.4	1.5	2.5%
82.4	93.7	86.2	99.0	96.2	84.7	90.4	6.8	7.5%
112.2	97.6	124.5	119.2	96.5	92.8	107.1	13.3	12.4%
				**Relative combined uncertainty for BFPD-IA:**				**6.4%**

File S3. Quality of samples.

Images of sample tubes indicating diversity in quality: (a) typical color of a Certified Reference Material (CRM), suitable for CRP immunoassay measurement and calibration; (b) non-hemolyzed sample, of similar visual status as the CRM, and of good quality to be measured with immunoassay methods; (c) non-hemolyzed, icteric sample, also judged to be of sufficient quality to perform the experiment; (d) hemolyzed sample, deviating color-wise from the CRM, and not suitable for measuring with immunoassay. We visually inspected all our samples and found them all to fall into visual status as (a), (b), or (c).

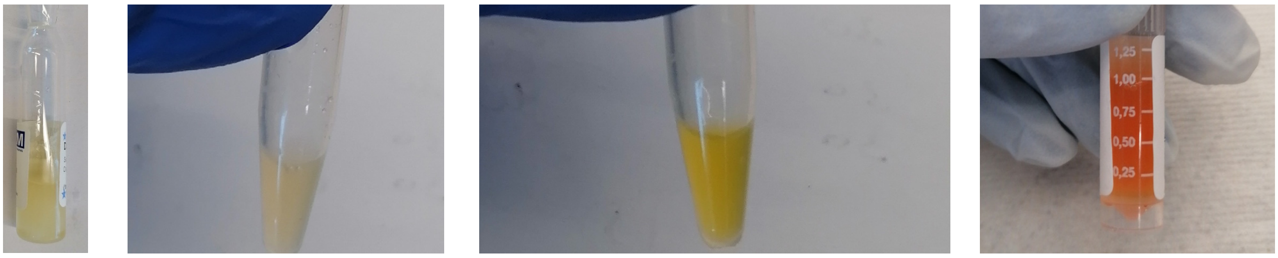

(**a**) (**b**) (**c**) (**d**)

File S4. CRP concentrations measured with BFPD-IA, ELISA, and ITA, and classification into zones.

CRP concentration values measured with the three different methods and accordingly qualitative classification into concentration zones A–D, for each scenario, described in manuscript Table 1. “ELISA”: Enzyme Linked Immunosorbent Assay. “ITA”: Immunoturbidimetric Assay. “BFPD”: Bound-Free Phase Detection Immunoassay. In the case of BFPD-IA, when the concentrations were calculated below or above the calibration curve limits, we denoted them “<LOD” and “>115,” respectively. The brackets in some zone classifications represent the secondary analysis mentioned in Section 2.4 and Section 3.2. According to this, one sample may be classified in either of two zones, taking into account the calculated relative combined uncertainty of the single measurement of each unknown sample. This uncertainty was calculated in Supplementary File S2 as ±1 standard deviation (“SD”) from the single measurement and is denoted as “min” and “max”.

**Sample #**	**Concentration (mg/L)**									**Scenario 1**			**Scenario 2**			**Scenario 3**			**Scenario 4**		
	**ELISA min (-1SD)**	**ELISA single**	**ELISA max (+1SD)**	**ITA min (-1SD)**	**ITA single**	**ITA max (+1SD)**	**BFPD min (-1SD)**	**BFPD single**	**BFPD max (+1SD)**	**ELISA**	**ITA**	**BFPD**	**ELISA**	**ITA**	**BFPD**	**ELISA**	**ITA**	**BFPD**	**ELISA**	**ITA**	**BFPD**
516-33	3.7	4.1	4.5	3.6	3.7	3.8	14.3	15.3	16.2	A	A	A	A	A	A	A	A	A	A	A	A
717-13	3.4	3.8	4.2	4.0	4.1	4.2	11.7	12.5	13.3	A	A	A	A	A	A	A	A	A	A	A	A
716-33	4.1	4.6	5.1	4.5	4.7	4.9	10.6	11.3	12.0	A	A	A	A	A	A	A	A	A	A	A	A
723-13	4.2	4.7	5.2	4.6	4.8	5.0	14.2	15.2	16.1	A	A	A	A	A	A	A	A	A	A	A	A
532-13	8.6	9.6	10.6	8.4	8.7	9.0	12.6	13.4	14.3	A	A	A	A	A	A	A	A	A	A	A	A
517-33	8.7	9.7	10.7	8.8	9.1	9.4	15.2	16.2	17.2	A	A	A	A	A	A	A	A	A	A	A	A
689-13	10.3	11.5	12.7	9.3	9.6	9.9	15.6	16.7	17.8	A	A	A	A	A	A	A	A	A	A	A	A
701-13	10.2	11.4	12.6	9.9	10.2	10.5	< LOD	< LOD	< LOD	A	A	A	A	A	A	A	A	A	A	A	A
708-13	14.0	15.6	17.2	13.9	14.4	14.9	17.5	18.7	19.9	A	A	A	A	A	A	A	A	A	A	A	A
714-13	14.6	16.2	17.8	18.2	18.8	19.4	18.2	19.4	20.6	A	A	A(B)	A	A	A(B)	A	A	A(B)	A	A	A(B)
709-13	18.7	20.8	22.9	19.0	19.7	20.4	23.3	24.9	26.5	B(A)	A(B)	B	B(A)	A(B)	B	B(A)	A(B)	B	B(A)	A(B)	B
729-13	16.6	18.5	20.4	19.2	19.9	20.6	18.0	19.2	20.4	A(B)	A(B)	A(B)	A(B)	A(B)	A(B)	A(B)	A(B)	A(B)	A(B)	A(B)	A(B)
704-13	19.9	22.1	24.3	20.5	21.2	21.9	21.4	22.8	24.3	B(A)	B	B	B(A)	B	B	B(A)	B	B	B(A)	B	B
460-33	14.2	15.8	17.4	21.8	22.5	23.2	18.8	20.1	21.4	A	B	B(A)	A	B	B(A)	A	B	B(A)	A	B	B(A)
567-23	17.9	19.9	21.9	24.3	25.1	25.9	21.4	22.8	24.3	A(B)	B	B	A(B)	B	B	A(B)	B	B	A(B)	B	B
503-23	24.1	26.8	29.5	24.6	25.4	26.2	25.3	27.0	28.7	B	B	B	B	B	B	B	B	B	B	B	B
455-23	29.3	32.6	35.9	26.9	27.8	28.7	32.0	34.1	36.3	B	B	B	B	B	B	B	B	B	B	B	B
515-23	21.5	23.9	26.3	31.4	32.5	33.6	23.3	24.9	26.5	B	B	B	B	B	B	B	B	B	B	B	B
702-23	31.6	35.2	38.8	32.7	33.8	34.9	29.3	31.3	33.3	B	B	B	B	B	B	B	B	B	B	B	B
758-23	27.2	30.3	33.4	34.5	35.7	36.9	29.2	31.2	33.1	B	B	B	B	B	B	B	B	B	B	B	B

769-13	33.7	37.5	41.3	34.7	35.9	37.1	34.2	36.5	38.8	B	B	B	B(C)	B	B	B	B	B	B	B	B
710-13	35.9	39.9	43.9	45.6	47.2	48.8	40.7	43.5	46.3	B	B	B	B(C)	C	C	B	B	B	B	B	B
727-33	38.7	43.0	47.3	47.0	48.6	50.2	44.4	47.5	50.5	B	B(C)	B(C)	C(B)	C	C	B	B	B	B	B	B
448-23	55.5	61.7	67.9	50.9	52.6	54.3	69.2	73.9	78.6	C	C	C	C	C	C	C(B)	B	C	B	B	B
501-23	54.4	60.5	66.6	56.6	58.5	60.4	75.1	80.2	85.3	C	C	C	C	C	C	C(B)	B(C)	C	B	B	B
457-23	45.4	50.5	55.6	56.9	58.8	60.7	57.3	61.2	65.1	C(B)	C	C	C	C	C	B	B(C)	C(B)	B	B	B
721-13	49.1	54.6	60.1	57.2	59.2	61.2	49.1	52.4	55.8	C(B)	C	C(B)	C	C	C	B(C)	B(C)	B	B	B	B
462-13	53.6	59.6	65.6	60.3	62.4	64.5	67.1	71.6	76.2	C	C	C	C	C	C	B(C)	C	C	B	B	B
465-23	52.5	58.4	64.3	61.7	63.8	65.9	61.9	66.1	70.4	C	C	C	C	C	C	B(C)	C	C	B	B	B
689-23	52.6	58.5	64.4	65.6	67.8	70.0	57.7	61.7	65.6	C	C	C	C	C	C	B(C)	C	C(B)	B	B	B
738-13	60.1	66.9	73.7	73.8	76.3	78.8	72.1	77.0	81.9	C	C	C	C	C	C	C	C	C	B	B	B
446-23	67.9	75.5	83.1	77.3	79.9	82.5	81.2	86.8	92.3	C	C	C	C	C	C	C	C	C	B	B	B
563-13	67.0	74.5	82.0	78.6	81.3	84.0	86.2	92.1	98.0	C	C	C	C	C	C	C	C	C	B	B	B
516-23	63.6	70.7	77.8	83.4	86.2	89.0	99.4	106.2	113.0	C	C	D(C)	C	C	C	C	C	C	B	B	C(B)
720-13	66.3	73.7	81.1	84.5	87.4	90.3	78.7	84.1	89.5	C	C	C	C	C	C	C	C	C	B	B	B
459-23	69.8	77.6	85.4	88.9	91.9	94.9	75.4	80.5	85.6	C	C	C	C	C	C	C	C	C	B	B	B
531-13	77.9	86.7	95.5	90.1	93.2	96.3	> 115	> 115	> 115	C	C	D	C	C	C	C	C	C	B	B	C
449-23	75.8	84.3	92.8	92.4	95.6	98.8	94.3	100.7	107.1	C	C	D(C)	C	C	C	C	C	C	B	B	C(B)
738-23	89.2	99.2	109.2	108.5	112.2	115.9	> 115	> 115	> 115	C(D)	D	D	C	C	C	C	C	C	B(C)	C	C
699-23	85.5	95.1	104.7	109.1	112.8	116.5	> 115	> 115	> 115	C(D)	D	D	C	C	C	C	C	C	B(C)	C	C
526-23	90.6	100.8	111.0	111.3	115.1	118.9	> 115	> 115	> 115	D(C)	D	D	C	C	C	C	C	C	C(B)	C	C
712-23	84.4	93.9	103.4	111.4	115.2	119.0	> 115	> 115	> 115	C(D)	D	D	C	C	C	C	C	C	B(C)	C	C
703-23	89.9	100.0	110.1	111.7	115.5	119.3	> 115	> 115	> 115	D(C)	D	D	C	C	C	C	C	C	C(B)	C	C
787-23	98.4	109.5	120.6	114.2	118.1	122.0	> 115	> 115	> 115	D(C)	D	D	C	C	C	C	C	C	C(B)	C	C
554-23	91.0	101.2	111.4	114.4	118.3	122.2	> 115	> 115	> 115	D(C)	D	D	C	C	C	C	C	C	C(B)	C	C
744-23	89.1	99.1	109.1	117.7	121.7	125.7	101.9	108.8	115.8	C(D)	D	D	C	C	C	C	C	C	B(C)	C	C
528-23	93.9	104.4	114.9	119.2	123.3	127.4	> 115	> 115	> 115	D(C)	D	D	C	C	C	C	C	C	C(B)	C	C
743-23	98.5	109.6	120.7	121.6	125.7	129.8	> 115	> 115	> 115	D(C)	D	D	C	C	C	C	C	C	C(B)	C	C
724-23	91.2	101.4	111.6	122.2	126.4	130.6	> 115	> 115	> 115	D(C)	D	D	C	C	C	C	C	C	C(B)	C	C
769-23	100.3	111.6	122.9	133.1	137.6	142.1	> 115	> 115	> 115	D	D	D	C	C	C	C	C	C	C	C	C
561-23	113.5	126.2	138.9	150.4	155.5	160.6	> 115	> 115	> 115	D	D	D	C	C	C	C	C	C	C	C	C
516-23x	103.9	115.6	127.3	157.4	162.8	168.2	> 115	> 115	> 115	D	D	D	C	C	C	C	C	C	C	C	C
762-23	125.1	139.1	153.1	216.2	223.6	231.0	> 115	> 115	> 115	D	D	D	C	C	C	C	C	C	C	C	C

File S5. Extended tables of correlation between methods and for each concentration zone.

The analysis takes into account possible classification of a sample near its neighboring zone due to the relative combined uncertainty of the single measurement, which is calculated according to Section 2.4 and Supplementary File S2. Any sample falling outside the colored boxes is considered a discrepancy.

**Scenario 1**	ITA Zone A	ITA Zone A/B or B/A	ITA Zone B	ITA Zone B/C or C/B	ITA Zone C	ITA Zone C/D or D/C	ITA Zone D
BFPD-IA Zone A	9						
BFPD-IA Zone A/B or B/A	1	1	1				
BFPD-IA Zone B		1	9				
BFPD-IA Zone B/C or C/B				1	1		
BFPD-IA Zone C					11		
BFPD-IA Zone C/D or D/C					2		
BFPD-IA Zone D					**1**		15

(a)

**Scenario 1**	ITA Zone A	ITA Zone A/B or B/A	ITA Zone B	ITA Zone B/C or C/B	ITA Zone C	ITA Zone C/D or D/C	ITA Zone D
ELISA Zone A	10		**1**				
ELISA zone A/B or B/A		2	2				
ELISA Zone B			7	1			
ELISA zone B/C or C/B					2		
ELISA Zone C					13		
ELISA zone C/D or D/C							11
ELISA Zone D							4

(b)

**Scenario 1**	BFPD-IA Zone A	BFPD-IA Zone A/B or B/A	BFPD-IA Zone B	BFPD-IA Zone B/C or C/B	BFPD-IA Zone C	BFPD-IA Zone C/D or D/C	BFPD-IA Zone D
ELISA Zone A	9	2					
ELISA zone A/B or B/A		1	3				
ELISA Zone B			7	1			
ELISA zone B/C or C/B				1	1		
ELISA Zone C					10	2	**1**
ELISA zone C/D or D/C							11
ELISA Zone D							4

(c)

**Scenario 2**	ITA Zone A	ITA Zone A/B or B/A	ITA Zone B	ITA Zone B/C or C/B	ITA Zone C
BFPD-IA Zone A	9				
BFPD-IA Zone A/B or B/A	1	1	1		
BFPD-IA Zone B		1	8		
BFPD-IA Zone B/C or C/B					
BFPD-IA Zone C					32

(d)

**Scenario 2**	ITA Zone A	ITA Zone A/B or B/A	ITA Zone B	ITA Zone B/C or C/B	ITA Zone C
ELISA Zone A	10		**1**		
ELISA zone A/B or B/A		2	2		
ELISA Zone B			5		
ELISA zone B/C or C/B			1		2
ELISA Zone C					30

(e)

**Scenario 2**	BFPD-IA Zone A	BFPD-IA Zone A/B or B/A	BFPD-IA Zone B	BFPD-IA Zone B/C or C/B	BFPD-IA Zone C
ELISA Zone A	9	2			
ELISA zone A/B or B/A		1	3		
ELISA Zone B			5		
ELISA zone B/C or C/B			1		2
ELISA Zone C					30

(f)

**Scenario 3**	ITA Zone A	ITA Zone A/B or B/A	ITA Zone B	ITA Zone B/C or C/B	ITA Zone C
BFPD-IA Zone A	9				
BFPD-IA Zone A/B or B/A	1	1	1		
BFPD-IA Zone B		1	10	1	
BFPD-IA Zone B/C or C/B				1	1
BFPD-IA Zone C			**1**	1	25

(g)

**Scenario 3**	ITA Zone A	ITA Zone A/B or B/A	ITA Zone B	ITA Zone B/C or C/B	ITA Zone C
ELISA Zone A	10		**1**		
ELISA zone A/B or B/A		2	2		
ELISA Zone B			8	1	
ELISA zone B/C or C/B			1	2	3
ELISA Zone C					23

(h)

**Scenario 3**	BFPD-IA Zone A	BFPD-IA Zone A/B or B/A	BFPD-IA Zone B	BFPD-IA Zone B/C or C/B	BFPD-IA Zone C
ELISA Zone A	9	2			
ELISA zone A/B or B/A		1	3		
ELISA Zone B			8	1	
ELISA zone B/C or C/B			1	1	4
ELISA Zone C					23

(i)

**Scenario 4**	ITA Zone A	ITA Zone A/B or B/A	ITA Zone B	ITA Zone B/C or C/B	ITA Zone C
BFPD Zone A	9				
BFPD-IA Zone A/B or B/A	1	1	1		
BFPD Zone B		1	22		
BFPD-IA Zone B/C or C/B			2		
BFPD Zone C			**1**		15

(j)

**Scenario 4**	ITA Zone A	ITA Zone A/B or B/A	ITA Zone B	ITA Zone B/C or C/B	ITA Zone C
ELISA Zone A	10		**1**		
ELISA zone A/B or B/A		2	2		
ELISA Zone B			23		
ELISA zone B/C or C/B					11
ELISA Zone C					4

(k)

**Scenario 4**	BFPD-IA Zone A	BFPD-IA Zone A/B or B/A	BFPD-IA Zone B	BFPD-IA Zone B/C or C/B	BFPD-IA Zone C
ELISA Zone A	9	2			
ELISA zone A/B or B/A		1	3		
ELISA Zone B			20	2	**1**
ELISA zone B/C or C/B					11
ELISA Zone C					4

(l)

File S6. Secondary analysis of the scatter plots including the relative combined uncertainty.

The analysis takes into account the calculation of the relative combined uncertainty and assigns error bars into all data points of the scatter plots of the manuscript Figure 2. The figures include red boxes that represent the cutoff values to show that some samples indeed lie at the border between zones and may legitimately be considered as falling into one or the other zone.

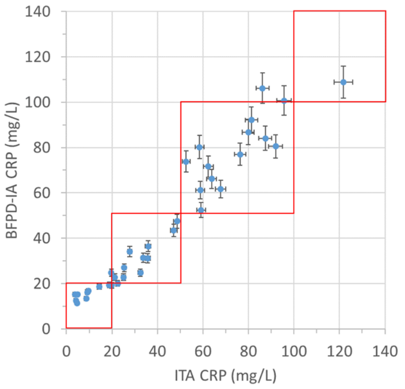

(a)
BFPD-IA vs ITA

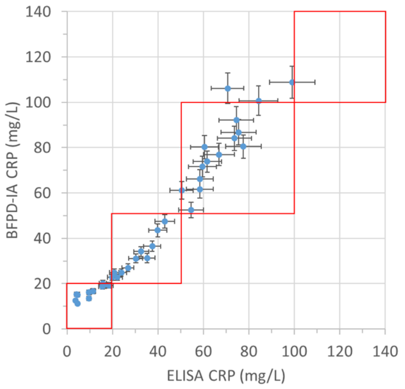

(b)
BFPD-IA vs ELISA

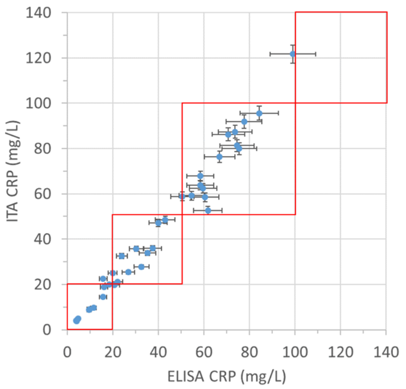

(c)
ITA vs ELISA

Scenario 2 (0, 20, 40 mg/L)

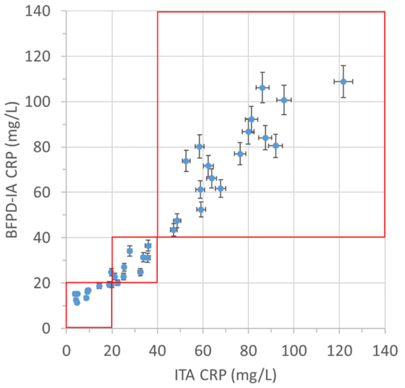

(d)
BFPD-IA vs ITA

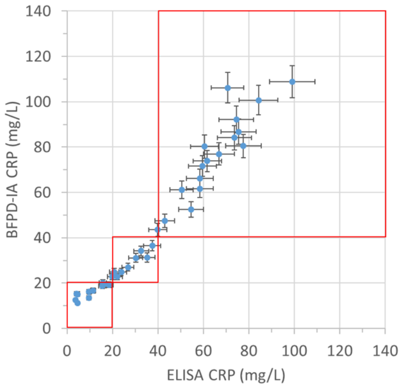

(e)
BFPD-IA vs ELISA

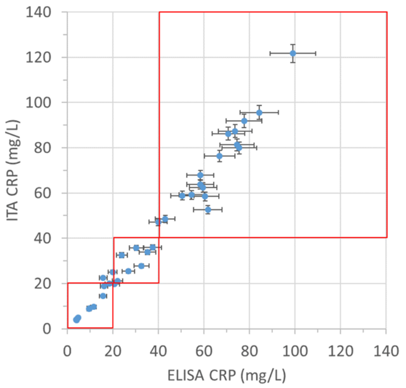

(f)
ITA vs ELISA

Scenario 3 (0, 20, 60 mg/L)

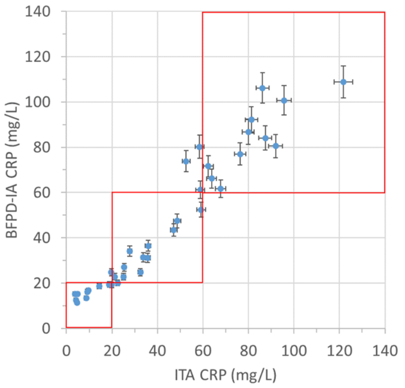

(g)
BFPD-IA vs ITA

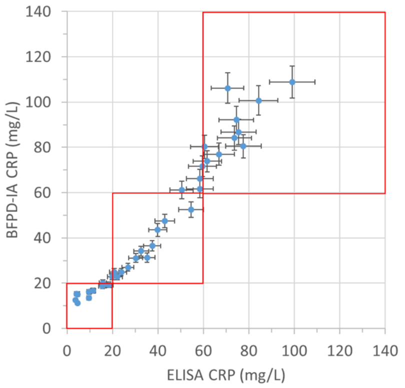

(h)
BFPD-IA vs ELISA

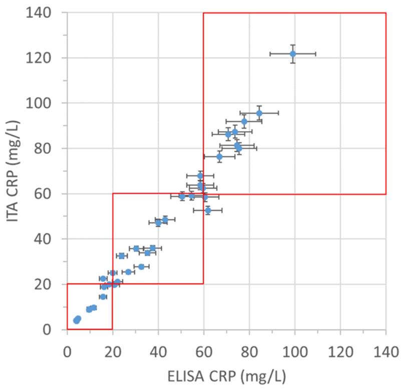

(i)
ITA vs ELISA

Scenario 4 (0, 50, 100 mg/L)

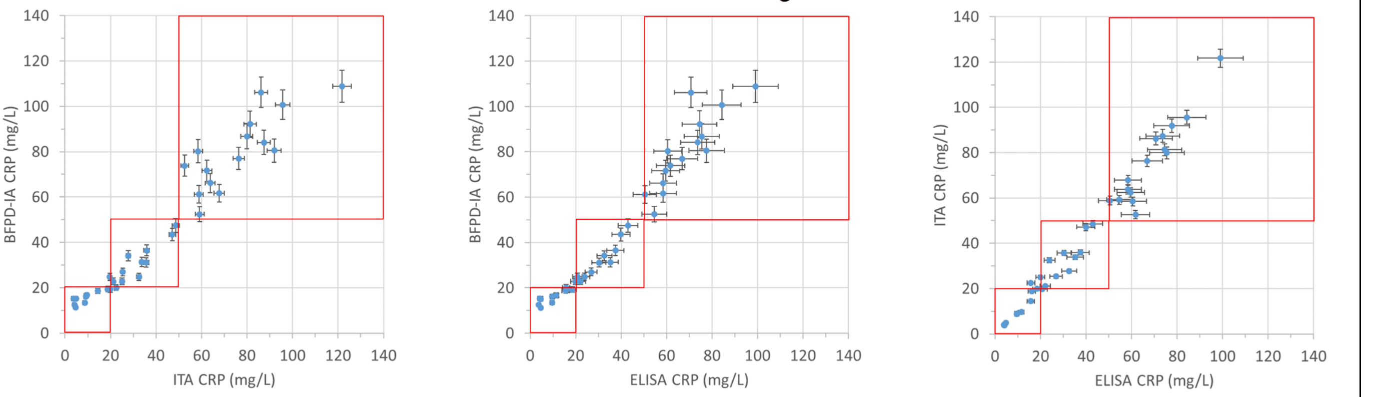

(j) BFPD-IA vs ITA

(k) BFPD-IA vs ELISA

(l) ITA vs ELISA
